# Optimising the Structure-Function Relationship at the Locus of Deficit in Retinal Disease

**DOI:** 10.3389/fnins.2019.00306

**Published:** 2019-04-09

**Authors:** Jack Phu, Michael Kalloniatis, Henrietta Wang, Sieu K. Khuu

**Affiliations:** ^1^Centre for Eye Health, The University of New South Wales, Kensington, NSW, Australia; ^2^School of Optometry and Vision Science, The University of New South Wales, Kensington, NSW, Australia

**Keywords:** glaucoma, retinitis pigmentosa, statokinetic dissociation, perimetry, visual neuroscience

## Abstract

Technologies such as optical coherence tomography have facilitated the visualization of anatomical tissues such as that of the retina. The availability of *in vivo* retinal anatomical data has led to the hypothesis that it may be able to accurately predict visual function from anatomical information. However, accurate determination of the structure-function relationship has remained elusive in part due to contributions of non-retinal sources of variability, thus imposing potential limitations in the fidelity of the relationship. Furthermore, differences in manifestation of functional loss due to different retinal loci of change (inner retina or outer retinal elements) have also been the subject of debate. Here, we assessed the application of a novel, more objective psychophysical paradigm to better characterize the relationship between functional and structural characteristics in the eye. Using ocular diseases with known loci of anatomical change (glaucoma, inner retinal loss; and retinitis pigmentosa, outer retinal loss), we compared conventional more subjective psychophysical techniques that may be contaminated by the presence of non-retinal sources of variability with our more objective approach. We show that stronger correlations between underlying retinal structure and visual function can be achieved across a breadth of anatomical change by using a more objective psychophysical paradigm. This was independent of the locus of structural loss (at the ganglion cells for glaucoma or photoreceptors for retinitis pigmentosa), highlighting the role of downstream retinal elements to serve as anatomical limiting factors for studying the structure-function relationship. By reducing the contribution of non-retinal sources of variability in psychophysical measurements, we herein provide a structure-function model with higher fidelity. This reinforces the need to carefully consider the psychophysical protocol when examining the structure-function relationship in sensory systems.

## Introduction

The relationship between anatomical structure and output function and behavior is a fundamental question in neuroscience, and understanding it provides important insight into how both biological markers mutually change due to processes such as ageing or disease (Weibel et al., 1991; Warrier et al., 2009; Canta et al., 2016). The eye is a biological structure in which the structure-function relationship can be readily studied (Harwerth et al., 2004, 2010; Harwerth and Quigley, 2006). Rather than necessarily requiring histological examination of the ocular tissues, downstream retinal elements in part responsible for visual perception are conducive to detailed *in vivo* examination using technologies such as optical coherence tomography (OCT) which provide comparable cross-sections of the retinal layers (Staurenghi et al., 2014). Visual functions such as contrast sensitivity can then be related to OCT results to examine the structure-function relationship (Medeiros et al., 2012; Raza et al., 2014; Asahina et al., 2017). Given their place within the visual pathway, linking propositions (Teller, 1984) therefore states that psychophysical data should be explained by the retinal substrates.

Despite developments in imaging technologies, limitations in the understanding of the structure-function relationship in the eye remain. Decreases in contrast sensitivity in aging and in disease have been well-documented (Heijl et al., 1987; Nowomiejska et al., 2016; Phu et al., 2017a,c, 2018c) with accompanying loss of structural tissue (Curcio and Allen, 1990; Curcio and Drucker, 1993; Curcio, 2001). However, discordance between the loci of structural and functional loss has been widely reported, where observable structural loss largely preceded measureable functional loss (Garway-Heath et al., 2000; Kerrigan-Baumrind et al., 2000), though in some cases functional loss may appear first (Marmor and Melles, 2014). Reasons for structure-function discordance in the eye have been vigorously debated, including selective changes in cell types, differentially affected visual pathways (described anatomically and psychophysically) or that it is part of the natural history of early stage disease (Turano and Wang, 1992; Anderson and O’brien, 1997; Owsley et al., 2000; Spry et al., 2005). Alternative test paradigms arising from these studies have not significantly improved the structure-function relationship in early disease (Jampel et al., 2011; Phu et al., 2017b).

A focal point of recent discussions in the structure-function relationship has therefore been the optimization of psychophysical stimulus parameters or statistical methods for analysis (Redmond et al., 2010, 2013; Mulholland et al., 2015; Phu et al., 2017c, 2018c; Rountree et al., 2018; Yoshioka et al., 2018). These studies to date have focussed on the nature of the stimulus, and have primarily examined pointwise, local structure-function correlations. A number of previous studies have used glaucoma as a model, as its structural locus of loss is well known, to test different statistical approaches for equating the structure-function relationship, including the linearising the measurement scale to either linear-linear or log-log units (Garway-Heath et al., 2000; Hood et al., 2007). These studies highlighted the need for topographically-matched comparisons, rather than relying upon global, averaged data.

Though pointwise topographically-matched structure-function relationships using static stimuli can be achieved using these manipulations (Malik et al., 2012), and recently to a very high level (*R*^2^ > 0.9) (Zangerl et al., 2019), the reliability of different functional measurement techniques in revealing the locus of structural loss along a topographical continuum of anatomical change remains less well-understood. Such changes typically occur within the visual field at the borders of localized retinal defects in which there may be a gradual decline in anatomical tissue, and consequently a gradual transition of visual function along the spatial extent of this continuum.

We have recently shown that a major source of test variability can be attributed to psychophysical technique employed for functional testing: whether the task is subjective or relatively more objective in which subjective factors such as response criterion are minimized. Subjective psychophysical tasks are confounded by the contribution of individual variability not necessarily attributable to the state of the underlying anatomical substrate (Phu et al., 2016a, 2018b). Although approaches to minimize subjective bias in laboratory-based psychophysical tasks have been well-established (Green and Swets, 1973), such techniques have not been widely adopted in clinical techniques. Differences in variability induced by the psychophysical technique have been recently exemplified by the work of Monter et al. (2017) who showed discordance between static and kinetic perimetry results in glaucoma. In combination, our work then led to the hypothesis that reducing non-retinal sources of variability in functional measurement using relatively more objective standard psychophysical procedures (Green and Swets, 1973; Gescheider, 1976) can lead to a more robust relationship across a spectrum of anatomical and functional change. Using our existing framework (Phu et al., 2018b), we hypothesized that altered sensitivity to different stimuli is not a unique feature of diseases, but has been confounded by an imprecise psychophysical method.

In the present study, we addressed these hypotheses by systematically examining psychophysical responses across a spectrum of anatomical change. Ocular diseases with different loci of structural change (glaucoma involving the inner retina, and retinitis pigmentosa involving primarily the outer retina) served as models for the structure-function relationship. We developed a technique to reduce typical individual biases in functional responses, thus providing a more accurate estimate of visual function. Consequently, we correlated structural and functional measurements across a spatial breadth of regions of anatomical change in disease in order to thoroughly map concordant changes to visual perception.

## Materials and Methods

### Participants

This study was carried out in accordance with the recommendations of the Human Research Ethics Committee at the University of New South Wales with written informed consent from all subjects. All subjects gave written informed consent in accordance with the Declaration of Helsinki. The protocol was approved by the Human Research Ethics Committee at the University of New South Wales. We have previously reported, in part, the some of the psychometric functions of a cohort of subjects with ocular diseases (Phu et al., 2018b), and we extracted a subset of results for analysis in the present study (6/7 subjects with glaucoma 4/5 subjects with retinitis pigmentosa), in addition to conducting further experiments and additional data collection (Supplementary Table S1). Seven subjects with primary open-angle glaucoma (median age 61.3 years, IQR: 56.5–68 years; 1 female, 6 males) and five subjects with retinitis pigmentosa (median age 58.2 years, IQR: 57–65 years; 2 females, 3 males) who had undergone comprehensive eye examination at the Centre for Eye Health, University of New South Wales comprised the cohort with ocular disease. Each subject had clinical findings characteristic of the ocular disease in question. Subjects with primary open-angle glaucoma had: characteristic optic nerve head changes with correlated thinning of the retinal nerve fiber layer, open and normal anterior chamber angles on gonioscopy, with or without elevated intraocular pressure, with corresponding retinotopic visual field loss (up to -12 dB mean deviation score). Subjects with retinitis pigmentosa had: characteristic fundus, electrophysiological and visual field changes, which included bone spicule patterns of hyperpigmentary retinal changes, thinning of particularly the outer retinal layers seen on OCT and corresponding reductions in the photopic and scotopic a- and b-waves (Whatham et al., 2014; Kalloniatis et al., 2016). Subjects with retinitis pigmentosa who had cystoid macular edema were excluded from the study (subjects with glaucoma had no macular changes). As the purpose was to compare the functional defects found using conventional perimetric techniques with the more objective laboratory-based test, we examined subjects with concordant structural and functional loss on clinical examination.

For this study, we also recruited five older subjects (median age 59.6 years, IQR: 51–67 years; 4 females, 1 male) for structural testing only. These subjects provided structural data for comparisons with the subjects with ocular disease to obtain difference plots with which to compare structural changes. The healthy subjects had also undergone comprehensive eye examinations at the Centre for Eye Health, and they all met the following criteria: best corrected visual acuity 20/30 or better; intraocular pressures < 21 mmHg; no anomalies of the macula, retina or optic nerve head; and normal standard automated perimetry and OCT results. To assess whether these subjects served as an adequate baseline for the comparisons, we compared their retinal thickness measurements with the work of Yoshioka et al. (2017), in which age-corrected normal ganglion cell thickness values were provided. We age-corrected the ganglion cell layer thickness values of the five prospectively recruited subjects to a 50 year-old equivalent subject, and compared these with the normative values of Yoshioka et al. (2017) The overall difference was 1.0 ± 3.0%, which suggests likely adequate representation of normal retinal thicknesses (Supplementary Figure S1).

For all subjects, spherical equivalent refractive errors were limited to between -6.00D and +6.00D, with cylinder power not exceeding -3.00DC. All subjects had best corrected visual acuities of 20/25 or better.

### Region of Interest for Structural and Functional Testing

We have previously reported on the region of interest for testing for the subjects with ocular diseases (Phu et al., 2018b). Briefly, to ensure that the structural and functional measurements could be reliably obtained using the laboratory-based instrumentation (see below), the region for testing in subjects with ocular disease had to meet the following criteria: the border of a scotoma between 3 and 27° of fixation (so sufficient step-wise range for laboratory-based testing could be achieved); it was a region with visual function measurable using a conventional Goldmann size III stimulus; and it had no cystoid macular or microcystic intraretinal changes that could confound measurement of retinal thickness. If multiple meridians met these criteria, it was chosen at random. For the age-matched controls, structural measurements were taken at the locations corresponding to the regions of interest of the subjects with ocular disease to obtain normal data for comparison. For all subjects, one eye was randomly selected to be tested, providing that it met the above criteria for the region of interest and study inclusion. The other eye was patched.

Three different approaches to measuring contrast sensitivity were compared within the chosen region of interest. The first two were subjective procedures–static contrast sensitivity thresholds and kinetic perimetry isopters–that have been primarily used in previous studies (Asahina et al., 2017; Yoshioka et al., 2018). Despite their prolific use, both provide a contrast sensitivity measurement that is confounded by individual criterion bias, and thus the resultant structure-function relationship is not exclusively reflective of function concordant with underlying anatomical structure. In comparison, the third approach was our proposed more objective psychophysical paradigm, which we hypothesize reduces the contribution of non-retinal components–i.e., individual sources of non-anatomical variability–of the structure-function relationship (Phu et al., 2016a,b). We have previously discussed these methods, but summarize them below.

### Functional Measurements of Contrast Sensitivity: (1) Static Perimetry Thresholds

**FIGURE 1:**
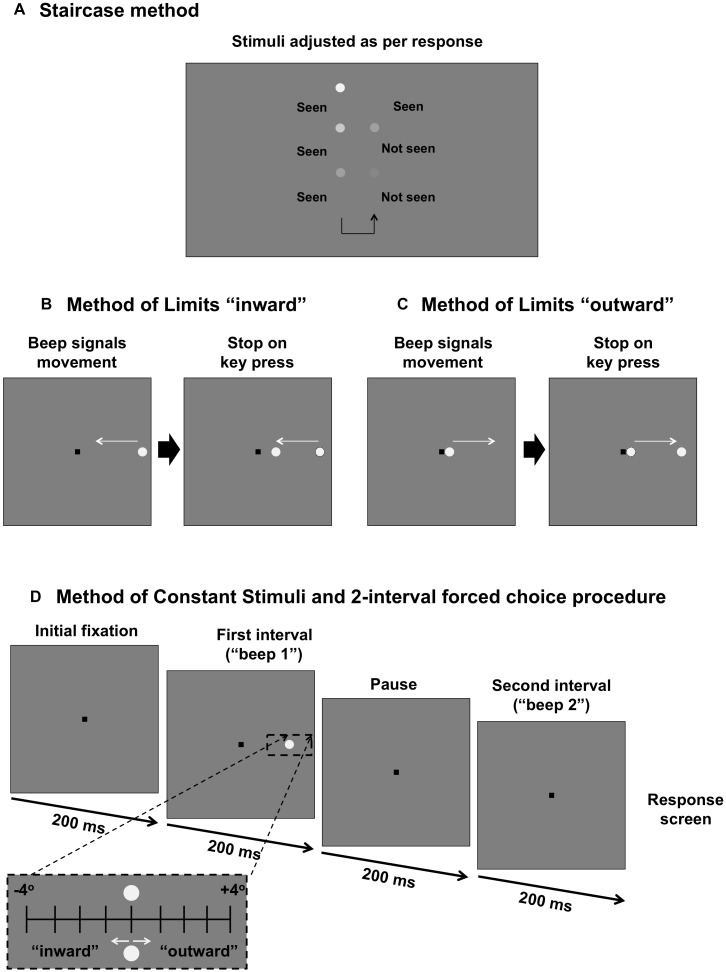
Comparison of psychophysical methods used in the present study. **(A)** Staircase procedure for determining sensitivity threshold. Stimulus intensity is modulated as per the response of the subject. **(B)** Method of Limits for determining the “inner” isopter. The black square is the fixation mark for the subject. A stimulus moves in an inward direction (straight path along the meridian) at a constant speed and the subject responds when they first see the target. **(C)** Method of Limits for determining the “outer” isopter. The stimulus moves outward along the meridian and the subject responds when the target disappears. **(D)** Method of Constant Stimuli and two interval forced choice procedure. The fixation mark is shown. After 200 ms, there is a tone signaling the first interval, followed by a 200 ms pause, then another tone signaling the second interval. After both intervals are shown, the fixation mark is shown again as the program waits for a response from the first subject. During one of the two intervals, a stimulus (static, inward moving or outward moving) is shown for 200 ms. The stimulus is randomly presented up to 4° inward or up to 4° outward in 1° steps around the midpoint of the isopters found in **(B)** and **(C)** (black dashed inset).

Static contrast sensitivity thresholds were measured using standard automated perimetry (Humphrey Field Analyzer in full threshold mode, Carl Zeiss Meditec, Dublin, CA, United States). It uses a thresholding procedure (two reversals, 4 dB–2 dB steps) to arrive at an approximate level of contrast sensitivity at fixed locations within the visual field (Figure 1A). Testing conditions were as per current clinical standards: Goldmann size III target (0.43° in diameter), presentation duration of 200 ms and a low photopic range background (10 cd.m^-2^), presented within both the 24–2 and 10–2 test grid for retinitis pigmentosa subjects (though the majority of retinitis pigmentosa subjects had visual field loss within the central 10° that were best resolved using the 10–2 grid) and within the 24–2 grid for glaucoma subjects. We determined the test location along the meridian of interest which first exhibited a statistically significant reduction in sensitivity commensurate with the disease process: contiguous points within the pattern deviation map at a significance level of *p* < 0.05 or lower (Mills et al., 2006). The location of the defect in degrees was then extracted as the eccentricity threshold.

### Functional Measurements of Contrast Sensitivity: (2) Kinetic Perimetry Isopters

The second method was a two-way Method of Limits, which was used to determine the perimetric isopter: the junction between seeing and non-seeing. Testing procedures resembled the conditions typically used and reported in clinical testing (0.43° circular increment stimulus presented upon a white-gray background of 10 cd.m^-2^). Stimuli were generated using custom written software (MATLAB version 7, Mathworks Inc., and Psychtoolbox version 3.0.11) and were presented upon a linearised iMac 27-inch computer driven at a frame rate of 60 Hz. A head and chin rest was used to ensure a constant viewing distance of 30 cm, and a trial frame with wide aperture lenses (38 mm) was used to correct for the subject’s refractive error and to compensate for the working distance.

To determine the kinetic perimetry isopters at the junction of the scotoma, the subject fixated upon a 0.6 × 0.6° black square (usually situated in the center of the screen, but sometimes offset to measure a more extensive field) of Weber contrast -0.2. While the subject fixated upon the fixation target, a tone signaled the onset of the stimulus. For the inner isopter (defined as the instance when the subject first sees a target moving from a region of non-seeing to seeing), the stimulus began in the far peripheral field and moved toward the square fixation target along the meridian of interest (Figure 1B). The subject was then asked to press a button on the keyboard to indicate when they first see the target. The outer isopter was determined by using a stimulus which began adjacent to the square fixation target, i.e., a region of seeing, before moving in the direction away from fixation along the same meridional path (Figure 1C). For the outward isopter, the subject was asked to indicate when the target first disappears.

For the subjects with retinitis pigmentosa, the contrast of the stimulus was the maximum output of the screen (approximately 375 cd.m^-2^). We used a stimulus with Weber contrast of 0.5 (equivalent to a 22 dB target on the Humphrey Field Analyzer) for testing in regions of deficit in subjects with glaucoma. For all conditions, the stimulus moved at a constant velocity of 4°/s. Each subject underwent five practice trials (each trial consisted of a response to one stimulus presentation) before recording commenced. Each subject supplied at least ten responses, recorded in degrees within visual space.

### Functional Measurements of Contrast Sensitivity: (3) Psychometric Functions

The third technique observes the well-established Method of Constant Stimuli which utilizes a two-interval forced choice procedure. We herein refer to this method as the more objective technique, in comparison to the staircase method and Method of Limits described above, as per established psychophysical principles. This was used to present stimuli at midpoint of the inner and outer isopters obtained from the Method of Limits, and then at 1° intervals inward and outward up to 4° either side along the meridian (nine eccentricity levels), as we have previously reported (Phu et al., 2016a, 2018b).

Figure 1D shows the process for testing using this paradigm. A tone would signal the first interval (200 ms). This would be followed by a blank interval of the same duration (200 ms), and then by a second tone to signal the second interval (200 ms). Then, the response screen, consisting solely of the fixation mark, would be shown as the program awaited the subject’s response. The stimulus appeared randomly in either the first or the second interval, and the subject was asked to indicate in which interval they saw the target; if they could not see it in either interval, they were asked to guess (thus the lower limit of the psychometric function was 50%). The next trial would start after the response. There were 10 trials per eccentricity level, and thus each run consisted of 90 trials in total. Each subject underwent one practice run.

The stimuli presented in this method could be static, moving (kinetic) inward or moving outward to provide a comparison for the first two methods using comparable static and moving stimuli. For the static condition, the stimulus was presented at the location for 200 ms. For the kinetic conditions, the stimulus was also shown for 200 ms, but moved either in an inward direction or outward direction at 4°/s. Conditions were tested separately, and each subject underwent testing at least twice for each condition. The proportion of times seen was recorded for each relative eccentricity value.

### Structural Measurements Using OCT

**FIGURE 2:**
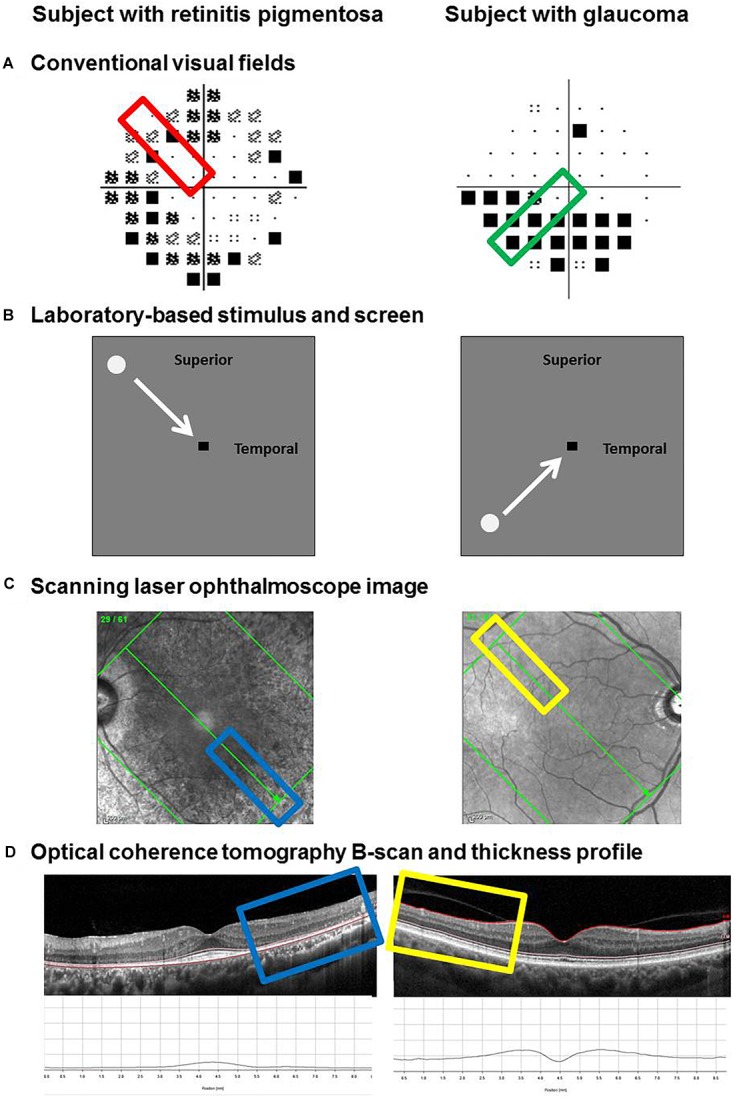
Method for determining and extracting retinal thickness measurements from functional regions of interest. Examples of two subjects are provided: one with retinitis pigmentosa (left) and one with glaucoma (right). Regions of interest were selected based on the clinical visual field result, marked by the colored rectangles **(A)**. The path of the visual stimulus during the laboratory-based phase of testing corresponded to the region of interest **(B)**. Retinal thickness measurements were taken along the retinotopically correspondent meridian using the Spectralis optical coherence tomography. The scanning laser ophthalmoscope image was used to line up the scan along the meridian of interest **(C)**. The individual B-scan of interest was manually segmented. From top to bottom, the inner limiting membrane (ILM), external limiting membrane (ELM) and Bruch’s membrane (BM) were used as the landmarks of interest to denote the inner retinal layers (ILM–ELM) and the outer retinal layers (ELM–BM). Thickness measurements were performed using the software’s thickness profile tool, highlighted by the colored rectangles **(D)**.

In addition to measuring functional responses, structural measurements were taken at the retinotopic location of the visual field defect given the selected region of interest (examples in Figure 2A). Measurements were obtained using OCT (Spectralis OCT, Heidelberg Engineering, Heidelberg, Germany). The posterior pole scan was used at an Automatic Real Time level of 36. The direction of the stimulus path (Figure 2B) guided the orientation of the scan grid (Figure 2C). For example, a stimulus that moved horizontally had a corresponding horizontal set of line scans. Although the instrument provides automatic segmentation of the retinal layers, we manually segmented the layers on the B-scan to account for potential segmentation errors such as those attributable to blood vessels (Figure 2D). Thickness measurements were taken at 1° intervals (∼288 microns) using the thickness profile tool of the software (Heidelberg Eye Explorer, Heidelberg Engineering, Heidelberg, Germany).

The results of individual subjects with ocular disease (inner retina from the inner limiting membrane to the external limiting membrane for glaucoma; and outer retina from the external limiting membrane to Bruch’s membrane for retinitis pigmentosa) were compared with the corresponding retinal thicknesses obtained at the same location in the five age-similar normal subjects. The difference in retinal thickness (in microns) was then plotted as a function of eccentricity (in degrees) for each subject.

### Statistical Analysis

Three approaches for testing concordance between structural and functional measurements were compared. In method 1, threshold eccentricities obtained using each technique were compared. We have previously described the method for determining threshold eccentricity (Phu et al., 2018b). Sigmoid nonlinear regression functions were fitted (GraphPad Prism version 7, La Jolla, CA, United States) to the structural and functional resultant data (*y*-axis), with eccentricity along the *x*-axis for each individual subject. The top of the sigmoid function was allowed to float between 0.9 and 1.0, to allow for a degree of false negative answers (Wichmann and Hill, 2001). Eccentricity threshold was defined as the EC50. The equations of the sigmoidal function are defined as: *y* = b + [(a-b) / (1 + 10^(logEC50-^*^x^*^)×HillSlope)^)], where *b* and *a* are the bottom and top values supplied by GraphPad Prism, respectively. These regression functions were performed on the normalized structural data (where 1.0 indicated the minimum amount of change and 0.0 indicating the maximum change) and the normalized proportions of stimuli seen when using our new paradigm (1.0 indicating the greatest proportion seen and 0.0 indicating the smallest proportion seen). The normalized structural and functional results were analyzed as a function of eccentricity within each individual and fitted using sigmoidal nonlinear regression functions as described above. We compared the area under the curve thus testing to determine whether there were systematic differences between structure and function or if concordance could be achieved. These functions were also compared using *F*-tests (all variables were included for comparison: top, bottom, logEC50 and HillSlope), with a significant effect adjusted using Bonferroni correction.

In method 2, using the above, we compared the difference in eccentricity thresholds between structure and the different functional techniques (subjective and our objective paradigm). A difference of 0 indicates co-localization of structure and function, assessed using a one-sample *t*-test.

In method 3, we were further able to correlate normalized structural and functional results when matched for test eccentricity. As with the above, the normalization process was conducted within individual, but for this analysis, the data was analyzed across the entire cohort. Non-parametric Spearman Rho was used examine the correlations, as per conventional statistics. In these analyses, the results were compared across subjects and between disease groups to test the hypothesis that discordance is not a feature unique to the disease process itself.

Since psychometric functions could not be obtained using conventional perimetric techniques (kinetic perimetry, Method of Limits, and standard automated perimetry, staircase), we only compared standard subjective techniques with the laboratory-based psychophysical method using the difference plot (method 2). The normalization process was not performed on the data obtained using Method of Limits or staircase procedures.

## Results

### Structural Eccentricity Thresholds

**FIGURE 3:**
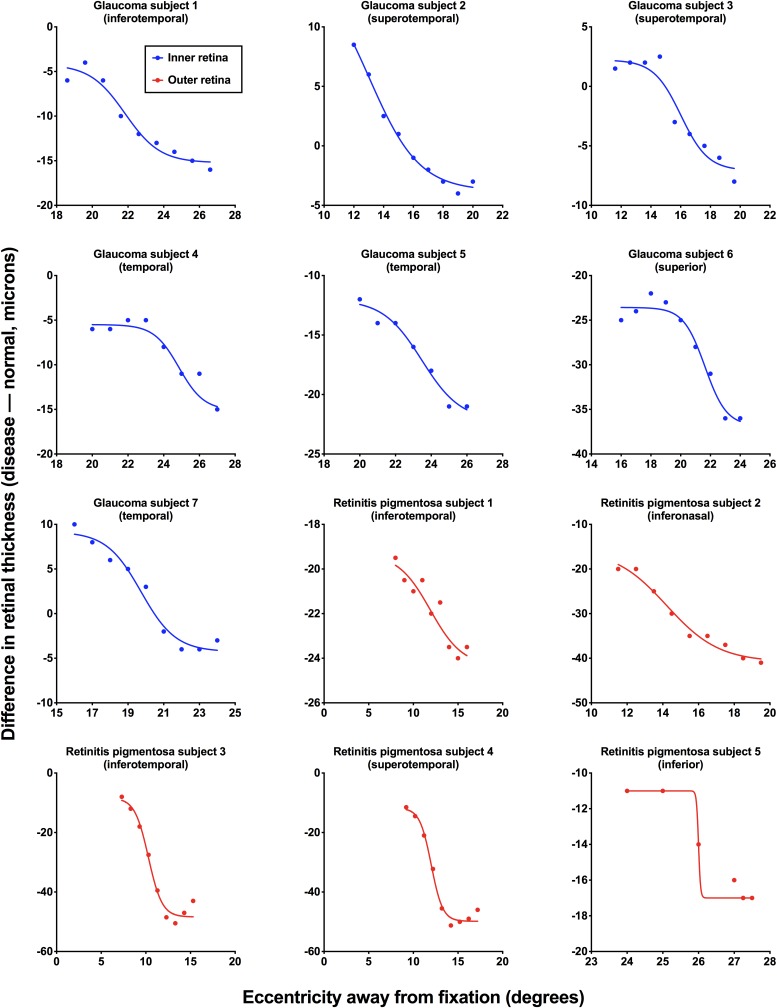
Individual structural functions for each subject with ocular disease. Difference in retinal thickness values [disease – normal (age-similar subjects), in microns] is plotted as a function of retinal eccentricity (away from fixation, in degrees). Inner retinal thicknesses were used for subjects with glaucoma (blue) and outer retinal thicknesses were used for subjects with retinitis pigmentosa (red). Each figure also shows the retinal test meridian (retinotopically correspondent with the functional test location).

For all subjects with ocular disease, we compared the structural measurements at their respective region of interest to the corresponding areas in the age-similar normal subjects. Figure 3 shows the results for individual patients with the difference in retinal thickness as a function of eccentricity. The standard deviation of residuals for all fits was 1.54° (interquartile range: 1.00°, 1.73°). Note that for some subjects the structural measurement intervals were < 1°, as these reached the limits of the scan grid, and we continued to use measurements from those intervals to set the top or floor of the function.

### Method 1–Comparison of Individual Normalized Structural and Functional Results

**FIGURE 4:**
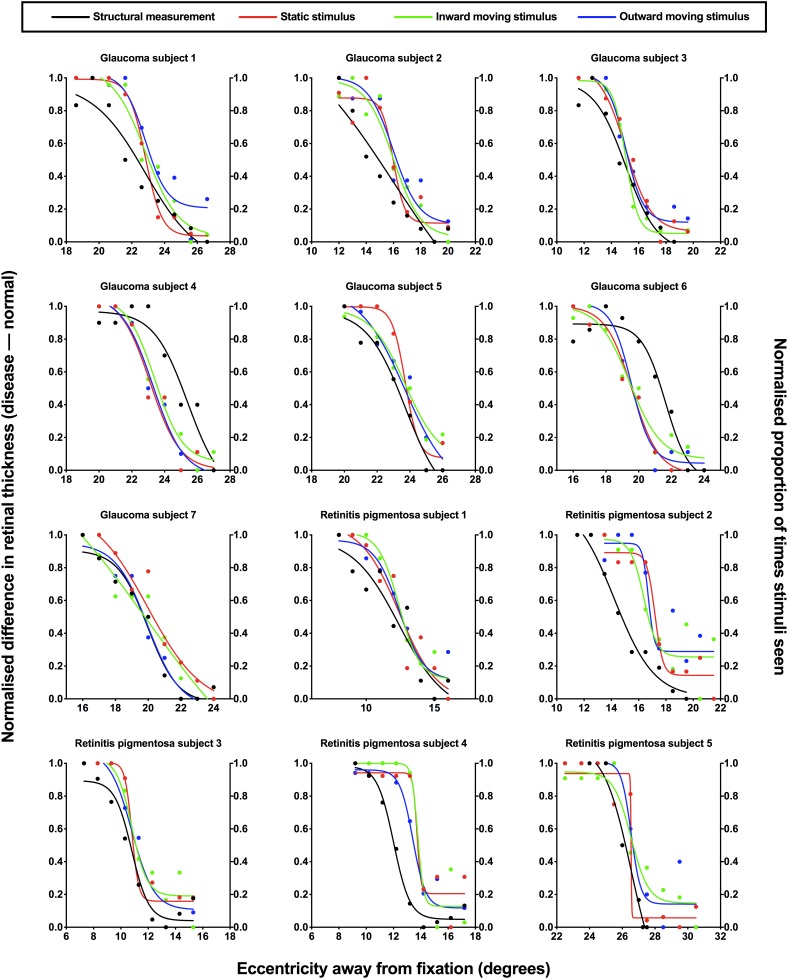
Normalized structural and functional measurements for each subject. Individual normalized structural (difference in retinal thickness, disease-normal; left *y*-axis) and functional (proportion of stimuli seen; right *y*-axis) measurements for each subject with ocular disease plotted as a function of test eccentricity (degrees). A normalized value of 1.0 indicates minimal difference in retinal thickness and a higher proportion of perception for structural and functional measurements, respectively, and vice versa for a normalized value of 0.0. Each subject has been labeled as per Figure 3, with the retinotopic test location omitted here for clarity. The difference colored points and lines indicate the condition: black, structural measurement; red, static stimulus for psychophysical testing; green, inward moving stimulus; blue, outward moving stimulus.

To provide direct comparison between structural and functional measurements, each subject’s structural and functional were normalized and the results were plotted against eccentricity for each subject (Figure 4). In normalizing results, both structural and functional results are expressed on the same scale, and as a measure of their fidelity, we could establish whether their relative change with location was the same. Two-way ANOVA showed a significant effect between structural and the three functional conditions [*F*(3,33) = 4.744, *p* = 0.0074], where the areas under the curve were lower for structure (median: 3.36, IQR: 2.95–3.82) compared to static (median: 4.13, IQR: 3.85–4.53), inward moving (median: 4.08, IQR: 3.76–4.63) and outward moving (median: 4.37, IQR: 3.71–4.63) stimuli. There was no effect of individual subject [*F*(11,33) = 1.019, *p* = 0.4522], suggesting a contribution of inter-individual variability as we have previously shown (Phu et al., 2018b). However, multiple comparisons showed that the difference in areas under the curve between structure and function were significant in two subjects (retinitis pigmentosa subject 4: structure–inward moving, *p* = 0.0472; and retinitis pigmentosa subject 5: structure–static, *p* = 0.0420; structure–inward moving, *p* = 0.0196; structure–outward moving, *p* = 0.0108). *F*-tests between functions within each individual was conducted in 11 subjects (retinitis pigmentosa subject 5’s data had slightly different ranges for structure and function) showed two subjects with statistically significant differences (glaucoma subject 6, *p* = 0.0025, and retinitis pigmentosa subject 4: *p* = 0.0003), but for all others, the top, bottom, slope and threshold parameters were not significantly different (average *F* value = 2.133, average *p* value = 0.1694).

### Method 2–Co-localization of Structural and Functional Eccentricity Thresholds

**FIGURE 5:**
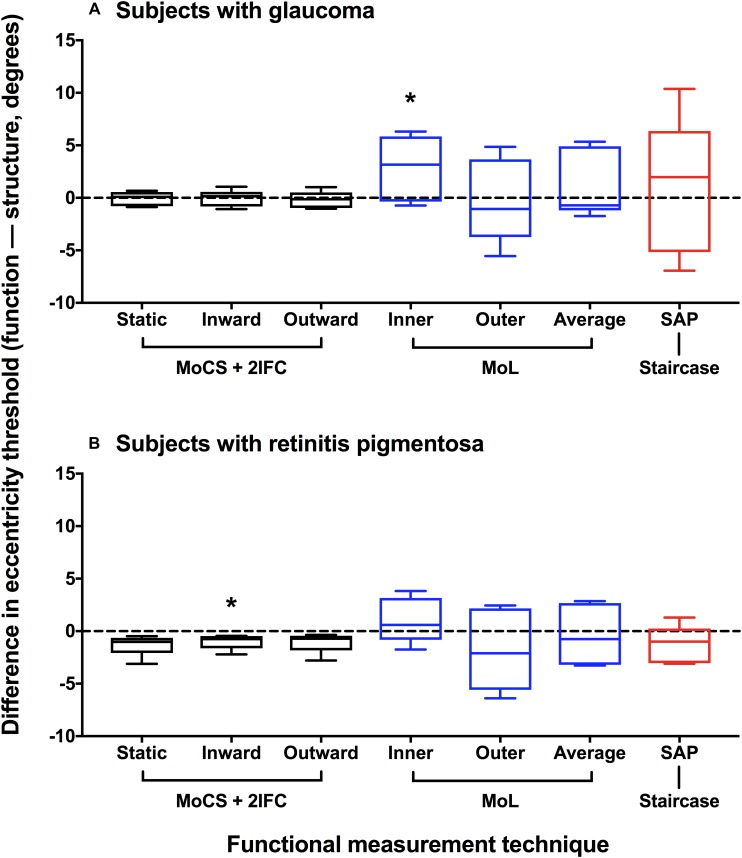
Difference plot of eccentricity threshold for each psychophysical method. Difference in eccentricity threshold (function–structure, degrees) for each disease group **(A)** glaucoma; **(B)** retinitis pigmentosa. A positive value indicates that the functional eccentricity threshold lay farther peripherally compared to the structural threshold. Method of Constant Stimuli (MoCS) and two interval forced choice (2IFC) procedure results (static, inward moving, and outward moving stimuli) are shown in black. Conventional perimetric measurement techniques are also shown: Method of Limits (MoL) results (inner and outer limits) in blue, and the staircase results using standard automated perimetry (SAP) in red. The black horizontal dashed line indicates no difference. The box and whisker plots indicate median, interquartile range and range. The asterisks indicate a result significantly different to 0 (one-sample *t*-test at a *p* < 0.05).

Based on the data presented in Figure 4, we also compared the differences in structural and functional eccentricity thresholds for all subjects in degrees (Figure 5). One sample *t*-test showed that the difference in eccentricity threshold was no different to 0 when using any of the functional measurement techniques for both ocular disease groups except for inward Method of Limits (*p* = 0.0302) for the glaucoma group and inner Method of Constant Stimuli for the retinitis pigmentosa group (*p* = 0.0379). However, the standard deviation of the eccentricity threshold differences was significantly greater when using all of the subjective techniques typically employed in clinical practice (Method of Limits such as in manual or semi-automated kinetic perimetry and staircase procedure typically used in static perimetry) compared to the two-interval forced choice procedure (glaucoma *p* = 0.0013; retinitis pigmentosa *p* = 0.0391), and was also greater than the standard deviation of the residuals for the structural functions. Although on average the functional measurements are co-localized with structural measurements, for each subject individual sources of non-retinal variability manifests as intra-subject mis-localization, or structure-function discordance.

### Method 3–Correlations of Normalized Structural and Functional Results Across All Subjects

**FIGURE 6:**
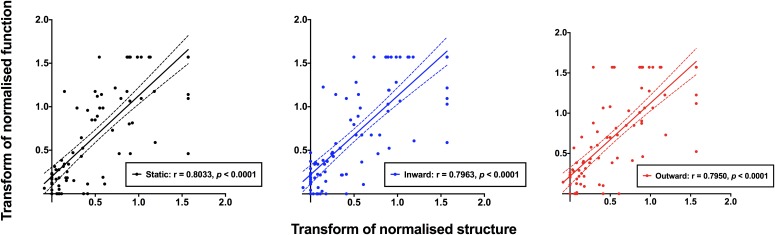
Normalized structural measurements plotted as a function of the corresponding normalized functional measurement extracted from Figure 5. Each datum point indicates a result from a single subject, with all subjects with ocular disease pooled together. Structural versus static (black), inward moving (blue) and outward moving (red) stimuli are shown separately. Due to the variability of structural and functional measurements, it was possible that some of the normalized results were less than 0.0 or greater than 1.0. Spearman Rho showed significant correlations between structure and function with all three conditions (*p* < 0.0001).

The normalized structural results were then plotted as a function of functional results, and Spearman Rho was used to determine the correlation after accounting for test eccentricity (Figure 6). All three relationships (structure versus static, inward moving or outward moving) were significant (*p* < 0.0001), with Spearman Rho values (95% confidence intervals) of 0.8201 (0.7378–0.8784), 0.8123 (0.7276–0.8727) and 0.8182 (0.7356–0.8786), respectively. The linear regression analysis showed slope values (standard error) of 0.8941 (0.0619), 0.8619 (0.0589) and 0.8655 (0.0595), respectively, and these were not significantly different to each other (*F* = 0.0859, *p* = 0.9177). The fits of the regression analysis (*R*^2^) were generally good at 0.6941, 0.6970, and 0.6949 for structure versus static, inward moving and outward moving, respectively.

## Discussion

The aim of the present study was to systematically characterize the structure-function relationship across a breadth of structural loss by using ocular diseases as a model for anatomical and perceptual change. Our work specifically exploits the ability of recent optical coherence tomography technologies in providing quantitative data of the ocular structures *in vivo*. For example, the inferences of losses of visual function at the borders of scotomas in a variety of retinal and post-retinal diseases using perimetric techniques have been studied by Enoch and colleagues, but correlations with anatomical structures were limited by technologies of the time (Enoch and Sunga, 1969; Enoch et al., 1970). In doing so, we wished to provide a psychophysical framework that could be adopted to reduce the contribution of non-retinal (or potentially non-anatomical in other sensory systems) factors that confound the structure-function relationship. We have shown that improvements in the structure-function relationship less affected by non-retinal sources of variability can be achieved by utilizing our more objective psychophysical paradigm, in line with our prediction (Green and Swets, 1973; Gescheider, 1976; Phu et al., 2018b). The robustness of this relationship persisted across the topographical breadth of anatomical change examined in the present cohort. As the robustness of the structure-function relationship appeared to be independent of the disease model, the discordance that is usually seen and reported is not unique or specific to the disease process, but instead is due to the psychophysical procedure itself.

### Subjectivity of Psychophysical Measurements and the Impact on the Structure-Function Relationship

We have previously discussed the significant individual variation present in functional measurements, whereby individual criterion bias contributes to trial-by-trial differences in results (Phu et al., 2016a, 2018b). The staircase method and Method of Limits are more subject to potential errors such as habituation and adaptation (Stewart and Hunt, 1993; Kunar et al., 2008), attention (Morales et al., 2015; Phu et al., 2016b, 2018a) or merely the individual’s own internal criterion bias (Hoskin et al., 2014; Mill et al., 2014) in comparison to Method of Constant Stimuli and forced choice procedures, which we referred to here as “more” objective psychophysical procedures. Though we recognize that subjective biases cannot be fully eliminated, the contributions of these errors therefore add a non-retinal component to the structure-function relationship, reducing its validity as an inference of behavior from underlying anatomical substrate.

The low variability and consistency across most subjects in our cohort suggested that the effect was robust. However, notably, some subjects with regions of interest farther away from the center showed greater variability, particularly in structural measurements. This is not surprising, due to the known difficulty in reliably obtaining measurements of ocular structure in the peripheral retina (Wenner et al., 2014). Though examination of a broad representation of stages of structural and functional deficits may be informative, we tested a breadth of structural integrity within each subject, and thus this already represents a potential spectrum of structural and functional alterations.

Another index of response variability that can be gleaned from functional data is the slope value of the psychometric function. We have previously reported on the slopes of some of these subjects (Phu et al., 2018b), and found that subjects with retinitis pigmentosa tended to have lower slope values compared to subjects with glaucoma. While this may be indicative of lower variability in subjects with retinitis pigmentosa, we have suggested that this may be due to the gradient of structural change which occurs differently between the diseases (Wyatt et al., 2007; Whatham et al., 2014). The structural data in the present study are additive to this, with significant overlaps in the functions between retinal thickness and psychophysical response data in Figure 4. We did not examine regions of unaffected retina in the subjects with ocular diseases in the present study, however, this would be informative in future studies.

### Accessibility of the Eye for Examining the Structure-Function Relationship: Structural Loci

Short of obtaining true histological samples (which also introduce confounders to the structure-function relationship), OCT provides an opportune surrogate measurement of retinal integrity (Chen et al., 2006). As our goal was to reduce the contribution of measurement variability, we used the total inner retinal thickness as the structural locus of glaucoma to maximize dynamic range, and because the nerve fiber layer and inner plexiform layer are known to be challenging to segment, especially in disease (Yoshioka et al., 2017, 2018). This becomes further complicated with progressive thinning of the inner retinal layers in the periphery (Wenner et al., 2014).

In retinitis pigmentosa, the outer retina is primarily affected and thus we used the outer retinal thickness as the locus of change (Rangaswamy et al., 2010; Battu et al., 2015). Though the inner retina is known to undergo remodeling in retinitis pigmentosa, the functional implications of such changes are not well-described (Kalloniatis et al., 2016). The reason for not extending this thickness to involve other overlying retinal layers is because unlike the inner retina, the outer retinal thickness does not typically change significantly beyond the central 10°.

Aside from OCT, increased accessibility to other nascent technologies could contribute to this discussion in the future. Specifically, adaptive optics OCT is an exciting technology that, compared to traditional OCT, can discern individual cell bodies, and can overcome assumptions made by spectral domain OCT regarding the cell density, distribution and integrity (Kocaoglu et al., 2011; Liu et al., 2017).

### Applications in Neuroscience Beyond Contrast Detection

Contrast detection is a fundamental task of the visual system, is simple to measure, and is a gateway to other visual tasks such as motion, form and stereopsis. In diseases, impairments in these dimensions have been suggested to indicate selective loss of specific visual pathways (Bosworth et al., 1997; McKendrick et al., 2005). Much like equating functional thresholds to static and moving targets, our paradigm for functional testing can also be readily applied to other visual functions beyond contrast detection to test this hypothesis.

Given the effects of other systemic diseases upon the eye, our paradigm could be extended to examine the structure-function relationship in other patient groups in order to facilitate early detection of the disease process and its associated functional sequelae. Examples of these include diabetic neuropathy (Neriyanuri et al., 2017) and Alzheimer’s disease (Cunha et al., 2016), which have garnered recent interest paralleling the increasing use of ocular imaging for detection of retinal loci of pathology. Again, our approach could be used to obtain a structure-function model that predicts eventual functional impairment.

So far, the present model has examined the detector elements in the eye which represent only the downstream components of the visual pathway. Potentially, our approach could provide insight into the structure-function relationship higher along the visual pathway, in areas specifically servicing visual functions such as color or motion in higher cortical areas, under conditions of anatomical change in aging and in disease. Neuroimaging techniques such as functional magnetic resonance imaging would be the next step to explore this relationship (Wandell et al., 1999; Gilaie-Dotan et al., 2013). Again, the contribution of our proposed method is to reduce the contribution of individual, potentially non-anatomical sources of variability such as criterion bias and decision-making.

### The Potential Effects of Eye Movements on the Structure-Function Relationship

One of the limitations of the present study was that no method of eye tracking was used for functional measurements. However, we predict that if fixation instability were to occur, then there would be an additional degree of discordance between structure and function. We used a typical criterion of unreliability of fixation losses of > 6° over 20% of the time during the Humphrey Field Analyzer test (Yoshioka et al., 2017, 2018) as a cut-off for all subjects. Using this criterion, we might expect a maximum discordance of 1.2° assuming no other significant eye movement for the observer’s result to be accepted as reliable. However, the standard deviation of the structure-function difference when using Method of Constant Stimuli and the forced choice procedure was less than this discordance, suggesting that eye movements did not play a significant role in the present study. The direction of movement would also be anticipated to be in the direction of the stimulus presentation, and thus a second prediction might be that the functional measurement would be biased in the direction away from fixation. However, no such bias was found, again supporting the notion that eye movements unlikely contributed to our results.

## Conclusion

We show that our paradigm of reducing the contribution of non-retinal–potentially non-anatomical–variability in behavioral responses to visual stimuli significantly improves the structure-function relationship across a broad range of anatomical change using models of outer retinal disease (retinitis pigmentosa) and inner retinal disease (glaucoma). Our results support the view of attentiveness to psychophysical method, rather than solely making alterations to stimulus parameters, to provide more robust estimations of visual function by reducing perceptual biases. These principles can be extended to visual tasks beyond simple contrast detection, and other sensory modalities. Our paradigm also has potential applications in other studies correlating behavior with anatomical structure in the neurosciences, and answers a crucial question regarding methods to examine output behavior that represent potentially otherwise poorly accessed or inaccessible anatomical substrates with high fidelity.

## Author Contributions

JP, HW, MK, and SK contributed to the design of the study and editing process. JP and HW contributed to data analysis. MK and SK provided materials for conducting the study. JP wrote the original draft of the manuscript.

## Funding

This work was supported by the National Health and Medical Research Council of Australia (NHMRC #1033224). Guide Dogs NSW/ACT are partners with the NHMRC grant.

## Conflict of Interest Statement

The authors declare that the research was conducted in the absence of any commercial or financial relationships that could be construed as a potential conflict of interest.

## Supplementary Material

The Supplementary Material for this article can be found online at: https://www.frontiersin.org/articles/10.3389/fnins.2019.00306/full#supplementary-material

## References

[B1] Anderson R. S. O’brien C. (1997). Psychophysical evidence for a selective loss of M ganglion cells in glaucoma. *Vision Res.* 37 1079–1083. 10.1016/S0042-6989(96)00260-X 9196726

[B2] Asahina Y. Kitano M. Hashimoto Y. Yanagisawa M. Murata H. Inoue T. (2017). The structure-function relationship measured with optical coherence tomography and a microperimeter with auto-tracking: the MP-3, in patients with retinitis pigmentosa. *Sci. Rep.* 7:15766 10.1038/s41598-017-16143-5 PMC5693920 29150681

[B3] Battu R. Khanna A. Hegde B. Berendschot T. T. Grover S. Schouten J. S. (2015). Correlation of structure and function of the macula in patients with retinitis pigmentosa. *Eye* 29 895–901. 10.1038/eye.2015.61 25952950 PMC4506342

[B4] Bosworth C. F. Sample P. A. Weinreb R. N. (1997). Perimetric motion thresholds are elevated in glaucoma suspects and glaucoma patients. *Vision Res.* 37 1989–1997. 10.1016/S0042-6989(96)00326-4 9274783

[B5] Canta A. Chiorazzi A. Carozzi V. A. Meregalli C. Oggioni N. Bossi M. (2016). Age-related changes in the function and structure of the peripheral sensory pathway in mice. *Neurobiol. Aging* 45 136–148. 10.1016/j.neurobiolaging.2016.05.014 27459934

[B6] Chen T. C. Cense B. Miller J. W. Rubin P. A. Deschler D. G. Gragoudas E. S. (2006). Histologic correlation of in vivo optical coherence tomography images of the human retina. *Am. J. Ophthalmol.* 141 1165–1168. 10.1016/j.ajo.2006.01.086 16765704

[B7] Cunha J. P. Moura-Coelho N. Proenca R. P. Dias-Santos A. Ferreira J. Louro C. (2016). Alzheimer’s disease: a review of its visual system neuropathology. Optical coherence tomography-a potential role as a study tool in vivo. *Graefes Arch. Clin. Exp. Ophthalmol.* 254 2079–2092. 10.1007/s00417-016-3430-y 27377656

[B8] Curcio C. A. (2001). Photoreceptor topography in ageing and age-related maculopathy. *Eye* 15 376–383. 10.1038/eye.2001.140 11450761

[B9] Curcio C. A. Allen K. A. (1990). Topography of ganglion cells in human retina. *J. Comp. Neurol.* 300 5–25. 10.1002/cne.903000103 2229487

[B10] Curcio C. A. Drucker D. N. (1993). Retinal ganglion cells in Alzheimer’s disease and aging. *Ann. Neurol.* 33 248–257. 10.1002/ana.410330305 8498808

[B11] Enoch J. M. Berger R. Birns R. (1970). A static perimetric technique believed to test receptive field properties: extension and verification of the analysis. *Doc. Ophthalmol.* 29 127–153. 10.1007/BF02346235 5313198

[B12] Enoch J. M. Sunga R. N. (1969). Development of quantitative perimetric tests. *Doc. Ophthalmol.* 26 215–229. 10.1007/BF00943979 5359517

[B13] Garway-Heath D. F. Caprioli J. Fitzke F. W. Hitchings R. A. (2000). Scaling the hill of vision: the physiological relationship between light sensitivity and ganglion cell numbers. *Invest. Ophthalmol. Vis. Sci.* 41 1774–1782. 10845598

[B14] Gescheider G. A. (1976). *Psychophysics: The Fundamentals.* England: Psychology Press.

[B15] Gilaie-Dotan S. Kanai R. Bahrami B. Rees G. Saygin A. P. (2013). Neuroanatomical correlates of biological motion detection. *Neuropsychologia* 51 457–463. 10.1016/j.neuropsychologia.2012.11.027 23211992 PMC3611598

[B16] Green D. M. Swets J. A. (1973). *Signal Detection Theory and Psychophysics.* Huntington, NY: Krieger Publishing.

[B17] Harwerth R. S. Carter-Dawson L. Smith E. L. III Barnes G. Holt W. F. Crawford M. L. (2004). Neural losses correlated with visual losses in clinical perimetry. *Invest. Ophthalmol. Vis. Sci.* 45 3152–3160. 10.1167/iovs.04-0227 15326134

[B18] Harwerth R. S. Quigley H. A. (2006). Visual field defects and retinal ganglion cell losses in patients with glaucoma. *Arch. Ophthalmol.* 124 853–859. 10.1001/archopht.124.6.853 16769839 PMC2265071

[B19] Harwerth R. S. Wheat J. L. Fredette M. J. Anderson D. R. (2010). Linking structure and function in glaucoma. *Prog. Retin. Eye Res.* 29 249–271. 10.1016/j.preteyeres.2010.02.001 20226873 PMC2878911

[B20] Heijl A. Lindgren G. Olsson J. (1987). Normal variability of static perimetric threshold values across the central visual field. *Arch. Ophthalmol.* 105 1544–1549. 10.1001/archopht.1987.01060110090039 3675288

[B21] Hood D. C. Anderson S. C. Wall M. Kardon R. H. (2007). Structure versus function in glaucoma: an application of a linear model. *Invest. Ophthalmol. Vis. Sci.* 48 3662–3668. 10.1167/iovs.06-1401 17652736

[B22] Hoskin R. Hunter M. D. Woodruff P. W. (2014). The effect of psychological stress and expectation on auditory perception: a signal detection analysis. *Br. J. Psychol.* 105 524–546. 10.1111/bjop.12048 25280122

[B23] Jampel H. D. Singh K. Lin S. C. Chen T. C. Francis B. A. Hodapp E. (2011). Assessment of visual function in glaucoma: a report by the american academy of ophthalmology. *Ophthalmology* 118 986–1002. 10.1016/j.ophtha.2011.03.019 21539982

[B24] Kalloniatis M. Nivison-Smith L. Chua J. Acosta M. L. Fletcher E. L. (2016). Using the rd1 mouse to understand functional and anatomical retinal remodelling and treatment implications in retinitis pigmentosa: a review. *Exp. Eye Res.* 150 106–121. 10.1016/j.exer.2015.10.019 26521764

[B25] Kerrigan-Baumrind L. A. Quigley H. A. Pease M. E. Kerrigan D. F. Mitchell R. S. (2000). Number of ganglion cells in glaucoma eyes compared with threshold visual field tests in the same persons. *Invest. Ophthalmol. Vis. Sci.* 41 741–748. 10711689

[B26] Kocaoglu O. P. Lee S. Jonnal R. S. Wang Q. Herde A. E. Derby J. C. (2011). Imaging cone photoreceptors in three dimensions and in time using ultrahigh resolution optical coherence tomography with adaptive optics. *Biomed. Opt. Express* 2 748–763. 10.1364/BOE.2.000748 21483600 PMC3072118

[B27] Kunar M. A. Flusberg S. Wolfe J. M. (2008). The role of memory and restricted context in repeated visual search. *Percept. Psychophys.* 70 314–328. 10.3758/PP.70.2.314 18372752

[B28] Liu Z. Kurokawa K. Zhang F. Lee J. J. Miller D. T. (2017). Imaging and quantifying ganglion cells and other transparent neurons in the living human retina. *Proc. Natl. Acad. Sci. U.S.A.* 114 12803–12808. 10.1073/pnas.1711734114 29138314 PMC5715765

[B29] Malik R. Swanson W. H. Garway-Heath D. F. (2012). ’Structure-function relationship’ in glaucoma: past thinking and current concepts. *Clin. Exp. Ophthalmol.* 40 369–380. 10.1111/j.1442-9071.2012.02770.x 22339936 PMC3693944

[B30] Marmor M. F. Melles R. B. (2014). Disparity between visual fields and optical coherence tomography in hydroxychloroquine retinopathy. *Ophthalmology* 121 1257–1262. 10.1016/j.ophtha.2013.12.002 24439759

[B31] McKendrick A. M. Badcock D. R. Morgan W. H. (2005). The detection of both global motion and global form is disrupted in glaucoma. *Invest. Ophthalmol. Vis. Sci.* 46 3693–3701. 10.1167/iovs.04-1406 16186351

[B32] Medeiros F. A. Zangwill L. M. Bowd C. Mansouri K. Weinreb R. N. (2012). The structure and function relationship in glaucoma: implications for detection of progression and measurement of rates of change. *Invest. Ophthalmol. Vis. Sci.* 53 6939–6946. 10.1167/iovs.12-10345 22893677 PMC3466074

[B33] Mill R. W. Alves-Pinto A. Sumner C. J. (2014). Decision criterion dynamics in animals performing an auditory detection task. *PLoS One* 9:e114076 10.1371/journal.pone.0114076 PMC4259461 25485733

[B34] Mills R. P. Budenz D. L. Lee P. P. Noecker R. J. Walt J. G. Siegartel L. R. (2006). Categorizing the stage of glaucoma from pre-diagnosis to end-stage disease. *Am. J. Ophthalmol.* 141 24–30. 10.1016/j.ajo.2005.07.044 16386972

[B35] Monter V. M. Crabb D. P. Artes P. H. (2017). Reclaiming the periphery: automated kinetic perimetry for measuring peripheral visual fields in patients with glaucoma. *Invest. Ophthalmol. Vis. Sci.* 58 868–875. 10.1167/iovs.16-19868 28159974

[B36] Morales J. Solovey G. Maniscalco B. Rahnev D. De Lange F. P. Lau H. (2015). Low attention impairs optimal incorporation of prior knowledge in perceptual decisions. *Atten. Percept. Psychophys.* 77 2021–2036. 10.3758/s13414-015-0897-2 25836765

[B37] Mulholland P. J. Redmond T. Garway-Heath D. F. Zlatkova M. B. Anderson R. S. (2015). Spatiotemporal summation of perimetric stimuli in early glaucoma. *Invest. Ophthalmol. Vis. Sci.* 56 6473–6482. 10.1167/iovs.15-16921 26447981

[B38] Neriyanuri S. Pardhan S. Gella L. Pal S. S. Ganesan S. Sharma T. (2017). Retinal sensitivity changes associated with diabetic neuropathy in the absence of diabetic retinopathy. *Br. J. Ophthalmol.* 101 1174–1178. 10.1136/bjophthalmol-2016-309641 28108570

[B39] Nowomiejska K. Brzozowska A. Koss M. J. Weleber R. G. Schiefer U. Rejdak K. (2016). Quantification of the visual field loss in retinitis pigmentosa using semi-automated kinetic perimetry. *Curr. Eye Res.* 41 993–998. 10.3109/02713683.2015.1079328 26470834

[B40] Owsley C. Jackson G. R. Cideciyan A. V. Huang Y. Fine S. L. Ho A. C. (2000). Psychophysical evidence for rod vulnerability in age-related macular degeneration. *Invest. Ophthalmol. Vis. Sci.* 41 267–273. 10634630

[B41] Phu J. Al-Saleem N. Kalloniatis M. Khuu S. K. (2016a). Physiologic statokinetic dissociation is eliminated by equating static and kinetic perimetry testing procedures. *J. Vis.* 16:5 10.1167/16.14.5 27829104

[B42] Phu J. Kalloniatis M. Khuu S. K. (2016b). The effect of attentional cueing and spatial uncertainty in visual field testing. *PLoS One* 11:e0150922 10.1371/journal.pone.0150922 PMC4777401 26937972

[B43] Phu J. Kalloniatis M. Khuu S. K. (2018a). Reducing spatial uncertainty through attentional cueing improves contrast sensitivity in regions of the visual field with glaucomatous defects. *Transl. Vis. Sci. Technol.* 7:8 10.1167/tvst.7.2.8 PMC5868861 29600116

[B44] Phu J. Kalloniatis M. Wang H. Khuu S. K. (2018b). Differences in static and kinetic perimetry results are eliminated in retinal disease when psychophysical procedures are equated. *Transl. Vis. Sci. Technol.* 7:22 10.1167/tvst.7.5.22 PMC6166892 30280007

[B45] Phu J. Khuu S. K. Bui B. V. Kalloniatis M. (2018c). A method using goldmann stimulus sizes i to v-measured sensitivities to predict lead time gained to visual field defect detection in early glaucoma. *Transl. Vis. Sci. Technol.* 7:17 10.1167/tvst.7.3.17 PMC5993363 29892496

[B46] Phu J. Khuu S. K. Nivison-Smith L. Zangerl B. Choi A. Y. J. Jones B. W. (2017a). Pattern recognition analysis reveals unique contrast sensitivity isocontours using static perimetry thresholds across the visual field. *Invest. Ophthalmol. Vis. Sci.* 58 4863–4876. 10.1167/iovs.17-22371 28973333 PMC5624776

[B47] Phu J. Khuu S. K. Yapp M. Assaad N. Hennessy M. P. Kalloniatis M. (2017b). The value of visual field testing in the era of advanced imaging: clinical and psychophysical perspectives. *Clin. Exp. Optom.* 100 313–332. 10.1111/cxo.12551 28640951 PMC5519947

[B48] Phu J. Khuu S. K. Zangerl B. Kalloniatis M. (2017c). A comparison of Goldmann III, V and spatially equated test stimuli in visual field testing: the importance of complete and partial spatial summation. *Ophthalmic Physiol. Opt.* 37 160–176. 10.1111/opo.12355 28211185 PMC5324678

[B49] Rangaswamy N. V. Patel H. M. Locke K. G. Hood D. C. Birch D. G. (2010). A comparison of visual field sensitivity to photoreceptor thickness in retinitis pigmentosa. *Invest. Ophthalmol. Vis. Sci.* 51 4213–4219. 10.1167/iovs.09-4945 20220048 PMC2910646

[B50] Raza A. S. Zhang X. De Moraes C. G. Reisman C. A. Liebmann J. M. Ritch R. (2014). Improving glaucoma detection using spatially correspondent clusters of damage and by combining standard automated perimetry and optical coherence tomography. *Invest. Ophthalmol. Vis. Sci.* 55 612–624. 10.1167/iovs.13-12351 24408977 PMC3908820

[B51] Redmond T. Anderson R. S. Russell R. A. Garway-Heath D. F. (2013). Relating retinal nerve fiber layer thickness and functional estimates of ganglion cell sampling density in healthy eyes and in early glaucoma. *Invest. Ophthalmol. Vis. Sci.* 54 2153–2162. 10.1167/iovs.12-10342 23439598

[B52] Redmond T. Garway-Heath D. F. Zlatkova M. B. Anderson R. S. (2010). Sensitivity loss in early glaucoma can be mapped to an enlargement of the area of complete spatial summation. *Invest. Ophthalmol. Vis. Sci.* 51 6540–6548. 10.1167/iovs.10-5718 20671278

[B53] Rountree L. Mulholland P. J. Anderson R. S. Garway-Heath D. F. Morgan J. E. Redmond T. (2018). Optimising the glaucoma signal/noise ratio by mapping changes in spatial summation with area-modulated perimetric stimuli. *Sci. Rep.* 8:2172 10.1038/s41598-018-20480-4 PMC5794745 29391459

[B54] Spry P. G. Johnson C. A. Mansberger S. L. Cioffi G. A. (2005). Psychophysical investigation of ganglion cell loss in early glaucoma. *J. Glaucoma* 14 11–19. 10.1097/01.ijg.0000145813.46848.b8 15650598

[B55] Staurenghi G. Sadda S. Chakravarthy U. Spaide R. F. and International Nomenclature for Optical Coherence Tomography (InOct) Panel (2014). Proposed lexicon for anatomic landmarks in normal posterior segment spectral-domain optical coherence tomography: the IN^∗^OCT consensus. *Ophthalmology* 121 1572–1578. 10.1016/j.ophtha.2014.02.023 24755005

[B56] Stewart W. C. Hunt H. H. (1993). Threshold variation in automated perimetry. *Surv. Ophthalmol.* 37 353–361. 10.1016/0039-6257(93)90065-F 8484168

[B57] Teller D. Y. (1984). Linking propositions. *Vision Res.* 24 1233–1246. 10.1016/0042-6989(84)90178-0 6395480

[B58] Turano K. Wang X. (1992). Motion thresholds in retinitis pigmentosa. *Invest. Ophthalmol. Vis. Sci.* 33 2411–2422. 1634338

[B59] Wandell B. A. Poirson A. B. Newsome W. T. Baseler H. A. Boynton G. M. Huk A. (1999). Color signals in human motion-selective cortex. *Neuron* 24 901–909. 10.1016/S0896-6273(00)81037-5 10624953

[B60] Warrier C. Wong P. Penhune V. Zatorre R. Parrish T. Abrams D. (2009). Relating structure to function: heschl’s gyrus and acoustic processing. *J. Neurosci.* 29 61–69. 10.1523/JNEUROSCI.3489-08.2009 19129385 PMC3341414

[B61] Weibel E. R. Taylor C. R. Hoppeler H. (1991). The concept of symmorphosis: a testable hypothesis of structure-function relationship. *Proc. Natl. Acad. Sci. U.S A.* 88 10357–10361. 10.1073/pnas.88.22.10357 1946456 PMC52927

[B62] Wenner Y. Wismann S. Preising M. N. Jager M. Pons-Kuhnemann J. Lorenz B. (2014). Normative values of peripheral retinal thickness measured with Spectralis OCT in healthy young adults. *Graefes Arch. Clin. Exp. Ophthalmol.* 252 1195–1205. 10.1007/s00417-013-2560-8 24514757

[B63] Whatham A. R. Nguyen V. Zhu Y. Hennessy M. Kalloniatis M. (2014). The value of clinical electrophysiology in the assessment of the eye and visual system in the era of advanced imaging. *Clin. Exp. Optom.* 97 99–115. 10.1111/cxo.12085 23865913

[B64] Wichmann F. A. Hill N. J. (2001). The psychometric function: I. Fitting, sampling, and goodness of fit. *Percept. Psychophys.* 63 1293–1313. 10.3758/BF03194544 11800458

[B65] Wyatt H. J. Dul M. W. Swanson W. H. (2007). Variability of visual field measurements is correlated with the gradient of visual sensitivity. *Vision Res.* 47 925–936. 10.1016/j.visres.2006.12.012 17320924 PMC2094527

[B66] Yoshioka N. Zangerl B. Nivison-Smith L. Khuu S. K. Jones B. W. Pfeiffer R. L. (2017). Pattern recognition analysis of age-related retinal ganglion cell signatures in the human eye. *Invest. Ophthalmol. Vis. Sci.* 58 3086–3099. 10.1167/iovs.17-21450 28632847 PMC5482244

[B67] Yoshioka N. Zangerl B. Phu J. Choi A. Y. J. Khuu S. K. Masselos K. (2018). Consistency of structure-function correlation between spatially scaled visual field stimuli and in vivo OCT ganglion cell counts. *Invest. Ophthalmol. Vis. Sci.* 59 1693–1703. 10.1167/iovs.17-23683 29610852

[B68] Zangerl B. Tong J. Alonso-Caneiro D. Yoshioka N. Kalloniatis M. (2019). *Clustered Spatial Alignment of Ganglion Cell Structure and Function Delivers Near Perfect Correlation Enabling Prediction of Visual Function.* Vancouver: ARVO.

