# Supplementary material for: Optimising the Structure-Function Relationship at the Locus of Deficit in Retinal Disease
Source: Front Neurosci. 2019 Apr 9;13:306. doi: 10.3389/fnins.2019.00306 (PMC6467237; doi:10.3389/fnins.2019.00306)
Supplement: Supplementary file 2 [file Data_Sheet_1.docx]

**Supplementary Material: Optimising the structure-function relationship at the locus of deficit in retinal disease**

Authors: Jack Phu^1,2*^, Michael Kalloniatis^1,2^, Henrietta Wang^1,2^, Sieu K. Khuu^2^

1. Centre for Eye Health, University of New South Wales, Kensington, NSW, Australia
2. School of Optometry and Vision Science, University of New South Wales, Kensington, NSW, Australia

**SUPPLEMENTARY FIGURE 1:** Following conversion from linear (microns) to logarithmic scale (dB) and age-correction to a 50 year-old equivalent using the age-correction factors provided by Yoshioka et al. (2017), a Bland-Altman analysis was performed on the difference in ganglion cell layer thickness (dB) between the present age-similar control cohort (*n* = 5) and the large (*n* = 201) normal cohort. Each datum point represents a test location from each of the 5 subjects in the present study. The black solid line indicates no difference, the red solid line indicates the bias from the Bland-Altman analysis and the red dotted lines indicate the 95% limits of agreement. Overall, there was no evidence of systematic bias, also as confirmed using an absolute difference plot as described in the main text (1.0 ± 3.0%).

**SUPPLEMENTARY TABLE 1:** Raw functional data for each subject with retinal disease in the present study for static, inward moving and outward moving stimuli (proportion seen as a function of eccentricity in degrees). Note that for retinitis pigmentosa subject 3, there was unreliable functional data for the static condition at location 11.3^o^, and hence that point has been excluded from analysis.
