# Supplementary figures and images for: Optimising the Structure-Function Relationship at the Locus of Deficit in Retinal Disease
Source: Front Neurosci. 2019 Apr 9;13:306. doi: 10.3389/fnins.2019.00306 (PMC6467237; doi:10.3389/fnins.2019.00306)

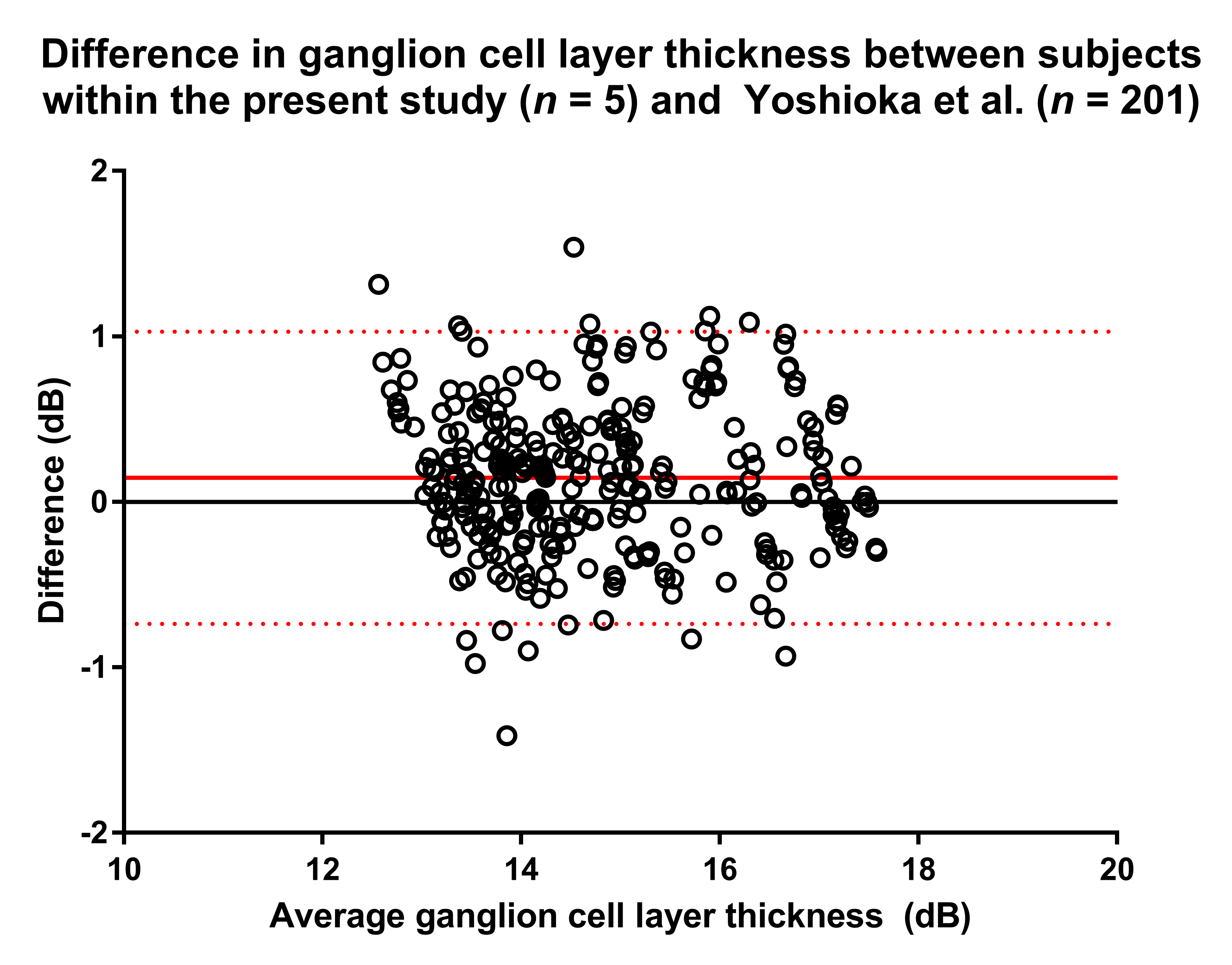

Supplement: Supplementary file 3 [file Image_1.TIF]
